# 18-Hydroxydolabella-3,7-diene synthase – a diterpene synthase from *Chitinophaga pinensis*

**DOI:** 10.3762/bjoc.13.171

**Published:** 2017-08-23

**Authors:** Jeroen S Dickschat, Jan Rinkel, Patrick Rabe, Arman Beyraghdar Kashkooli, Harro J Bouwmeester

**Affiliations:** 1 Kekulé-Institute of Organic Chemistry and Biochemistry, University of Bonn, Gerhard-Domagk-Straße 1, 53121 Bonn, Germany; 2 Laboratory of Plant Physiology, Wageningen University, Droevendaalsesteeg 1, 6708 PB Wageningen, The Netherlands,; 3 Swammerdam Institute for Life Sciences, University of Amsterdam, Sciencepark 904, 1098 XH Amsterdam, The Netherlands

**Keywords:** biosynthesis, *Chitinophaga pinensis*, *Nicotiana benthamiana*, structure elucidation, terpenes

## Abstract

The product obtained in vitro from a diterpene synthase encoded in the genome of the bacterium *Chitinophaga pinensis*, an enzyme previously reported to have germacrene A synthase activity during heterologous expression in *Escherichia coli*, was identified by extensive NMR-spectroscopic methods as 18-hydroxydolabella-3,7-diene. The absolute configuration of this diterpene alcohol and the stereochemical course of the terpene synthase reaction were addressed by isotopic labelling experiments. Heterologous expression of the diterpene synthase in *Nicotiana benthamiana* resulted in the production of 18-hydroxydolabella-3,7-diene also in planta, while the results from the heterologous expression in *E. coli* were shown to be reproducible, revealing that the expression of one and the same terpene synthase in different heterologous hosts may yield different terpene products.

## Introduction

**Scheme 1:**
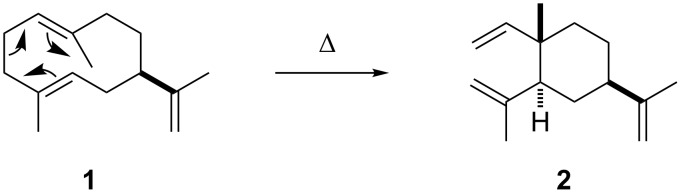
Germacrene A (**1**) and its Cope rearrangement to β-elemene (**2**).

Terpene synthases convert a handful of simple linear and achiral oligoprenyl diphosphates in just one enzymatic step into a remarkable diversity of usually polycyclic structurally complex lipophilic terpenes with multiple stereogenic centres. In their active sites type I terpene synthases contain the highly conserved aspartate-rich motif DDXX(X)(D,E) and the NSE triad NDXXSXX(R,K)(E,D), modified to a DTE triad in plants, for binding of the Mg^2+^ cofactor that forms a trinuclear (Mg^2+^)_3_ cluster to which the diphosphate portion of the substrate binds. Upon substrate binding the active site closes, resulting in hydrogen bonds between the substrate’s diphosphate and the pyrophosphate sensor, a highly conserved arginine located 43 amino acids upstream of the NSE triad, and the RY dimer, a highly conserved motif at the C-terminus. The substrate is ionised by extrusion of diphosphate, yielding a highly reactive allyl cation that can react in a cyclisation cascade by attack of olefinic double bonds to the cationic centre, hydride shifts and Wagner–Meerwein rearrangements. The process is usually terminated by deprotonation or attack of water to yield a lipophilic terpene hydrocarbon or alcohol. Among the first investigated terpene synthases were the (+)- and (−)-bornyl diphosphate synthases from the plants *Salvia officinalis* and *Tanacetum vulgare* forming a more polar product by the unusual termination via reattack of diphosphate [1], the trichodiene synthase from the fungus *Trichothecium roseum* [2], and pentalenene synthase from *Streptomyces exfoliatus* [3]. Recently, the first terpene synthases were reported from a eukaryotic soil microorganism, the social amoeba *Dictyostelium discoideum* [4–5]. With respect to bacterial enzymes, many terpene synthases have been identified and their products have been structurally characterised (reviewed in [6], following reports: [7–14]). One possible method to investigate the products of terpene synthases is the expression of terpene synthase genes in a heterologous host, as was recently performed for a large number of bacterial enzymes in an engineered *Streptomyces avermitilis* strain from which the biosynthesis genes for all other natural products were deleted, allowing a relatively easy purification of the terpene synthase products from culture extracts [15–16]. The heterologous expression of terpene synthase genes in *Escherichia coli* is also frequently successful, resulting in the production of volatile terpenes by this bacterium that can be detected in headspace extracts [17–18]. In one of these previous reports [17] we have described a terpene synthase from *Chitinophaga pinensis* DSM 2588 (accession number WP_012789469) as a sesquiterpene synthase for germacrene A (**1**), which was based on the identification of this compound and its Cope rearrangement product β-elemene (**2**) formed by the thermal impact during GC–MS analysis [19] in *E. coli* headspace extracts under heterologous expression of the terpene synthase gene (Scheme 1). Here we present the diterpene synthase activity of this enzyme in in vitro experiments and the first heterologous expression of a bacterial terpene synthase gene in a plant, *Nicotiana benthamiana*.

## Results and Discussion

### Characterisation of a diterpene synthase from *Chitinophaga pinensis* in vitro

**Figure 1:**
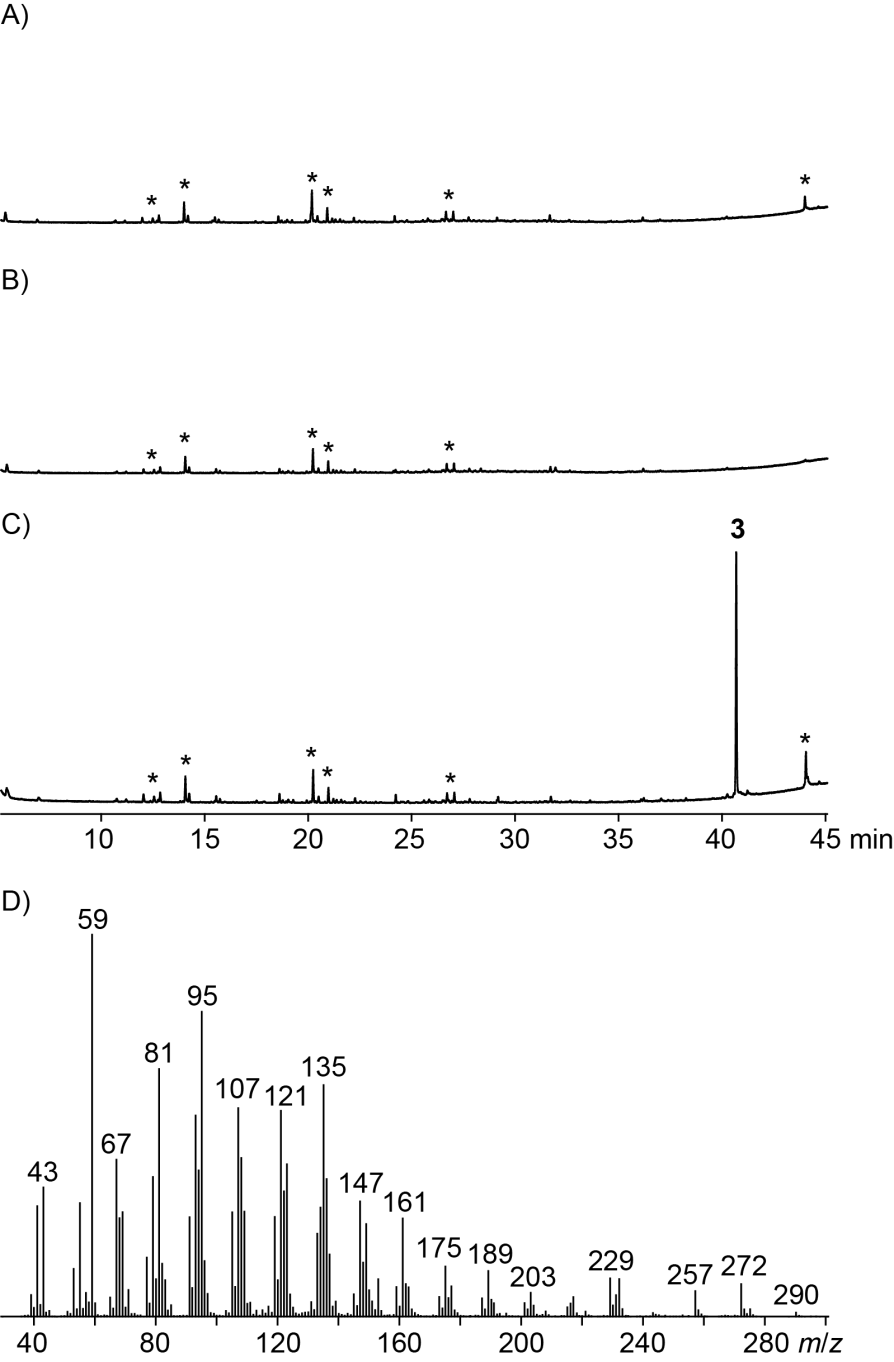
In vitro terpene synthase activity of the investigated recombinant enzyme from *C. pinensis*, showing no formation of monoterpenes from GPP (A) and no formation of sesquiterpenes from FPP (B), but formation of a single diterpene alcohol **3** from GGPP (C) with the mass spectrum depicted in (D). Asterisks indicate non-terpenoid contaminants such as plasticisers.

The terpene synthase from *C. pinensis* was heterologously expressed in *E. coli* as a recombinant protein with a C-terminal polyhistidine tag using a previously reported pET28c-based expression construct [17] and purified by Ni-NTA affinity chromatography (Figure S1, Supporting Information File 1). The purified enzyme was tested in in vitro experiments for mono-, sesqui- and diterpene activity by incubation with geranyl (GPP), farnesyl (FPP) and geranylgeranyl diphosphate (GGPP) as substrates, which yielded a single product **3** only from GGPP, but no products from FPP and GPP as demonstrated by GC–MS analysis (Figure 1). The mass spectrum of **3** showed a molecular ion at *m*/*z* = 290 pointing to a diterpene alcohol and a base peak ion at *m*/*z* = 59 indicative of a 2-hydroxyisopropyl group that frequently occurs in terpene alcohols. Both findings, i.e., no production of sesquiterpenes from FPP in in vitro experiments with recombinant purified enzyme as well as the emission of sesquiterpenes by *E. coli* during heterologous expression, were fully reproducible (Figure S2, Supporting Information File 1).

**Scheme 2:**
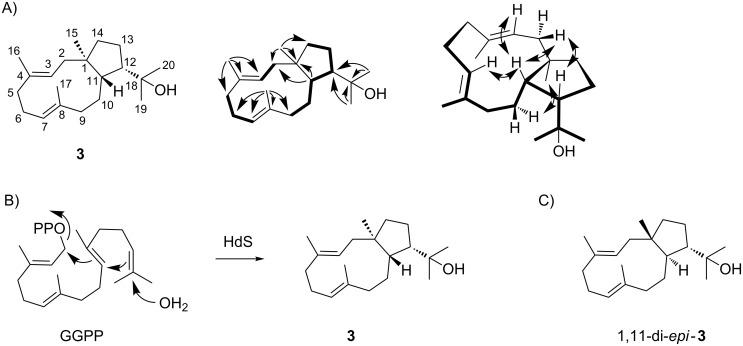
Product obtained from the diterpene synthase from *C. pinensis*. (A) Structure of (1*R*,3*E*,7*E*,11*S*,12*S*)-18-hydroxydolabella-3,7-diene (**3**), contiguous ^1^H,^1^H-COSY spin systems (bold), and diagnostic HMBC and NOESY correlations (single and double headed arrows). (B) Cyclisation mechanism for the conversion of GGPP into **3** by HdS. (C) Structure of the known stereoisomer 1,11-di-*epi*-**3**.

The compound **3** obtained from the in vitro incubation of GGPP was purified and its structure was elucidated by extensive one- and two-dimensional NMR spectroscopic methods (Table 1, Figures S3–S9, Supporting Information File 1). The ^13^C NMR spectrum showed five signals for methyl groups, seven aliphatic CH_2_ groups, two aliphatic and two olefinic CH groups, and four signals for quarternary carbons including one connected to oxygen and two olefinic carbons, suggesting the structure of a bicyclic diterpene alcohol. The ^1^H,^1^H-COSY spectrum revealed three contiguous spin systems for C2–C3, C5–C6–C7, and C9–C10–C11–C12–C13–C14 (Scheme 2). Key HMBC correlations from H19 and H20 to C12 and C18 placed the 2-hydroxyisopropyl group at C12, while HMBC correlations from H17 to C6, C7, C8 and C9 located the C8–C17 fragment between C7 and C9. HMBC crosspeaks between H16 and C3, C4 and C5 indicated the C3–C4–C5 connection, and HMBC correlations between H15 and C1, C2 and C14, and between H11, C1 and C2 established the bonds between the quarternary carbon C1 and its four neighbours. Diagnostic NOESY correlations between H11 and H2β, H3 and H7, between H12 and H2β, and between H10α and H15 established the relative configuration of **3**, resulting in the structure of (1R*,3*E*,7*E*,11S*,12S*)-18-hydroxydolabella-3,7-diene and identifying the terpene synthase from *C. pinensis* as 18-hydroxydolabella-3,7-diene synthase (HdS).

**Table 1:** NMR data of **3** recorded in C_6_D_6_.

C^a^	^13^C (δ)^b^	^1^H (δ, m, *J*, int)^c^

1	47.5 (C_q_)	–
2	42.6 (CH_2_)	2.19 (m, 1H, Hβ) 1.71 (dd, *J* = 6.2, *J* = 13.8, 1H, Hα)
3	126.5 (CH)	5.16 (dd, *J* = 9.7, *J* = 5.8, 1H)
4	134.0 (C_q_)	–
5	40.2 (CH_2_)	2.12 (m, 1H) 2.06 (m, 1H)
6	25.0 (CH_2_)	2.22 (m, 1H, Hβ) 2.05 (m, 1H, Hα)
7	128.2 (CH)	4.87 (dd, *J* = 10.0, *J* = 4.3, 1H)
8	134.0 (C_q_)	–
9	39.2 (CH_2_)	2.27 (m, 1H, Hα) 2.14 (m, 1H, Hβ)
10	23.7 (CH_2_)	2.13 (m, 1H, Hβ) 1.23 (m, 1H, Hα)
11	42.1 (CH)	1.84 (m, 1H)
12	53.7 (CH)	1.84 (ddd, *J* = 10.4, *J* = 7.4, *J* = 7.4, 1H)
13	26.0 (CH_2_)	1.53 (m, 1H) 1.53 (m, 1H)
14	41.3 (CH_2_)	1.47 (m, 1H, Hα) 1.39 (m, 1H, Hβ)
15	24.9 (CH_3_)	1.08 (s, 3H)
16	16.6 (CH_3_)	1.59 (s, 3H)
17	16.0 (CH_3_)	1.47 (s, 3H)
18	72.1 (C_q_)	–
19	30.8 (CH_3_)	1.11 (s, 3H)
20	30.7 (CH_3_)	1.18 (s, 3H)

^a^Carbon numbering as shown in Scheme 2. ^b^Chemical shifts δ in ppm and assignment of carbons by ^13^C-DEPT135 spectroscopy. ^c^Chemical shifts δ in ppm, multiplicity m (s = singlet, d = doublet, t = triplet, m = multiplet), coupling constants *J* are given in Hertz.

**Figure 2:**
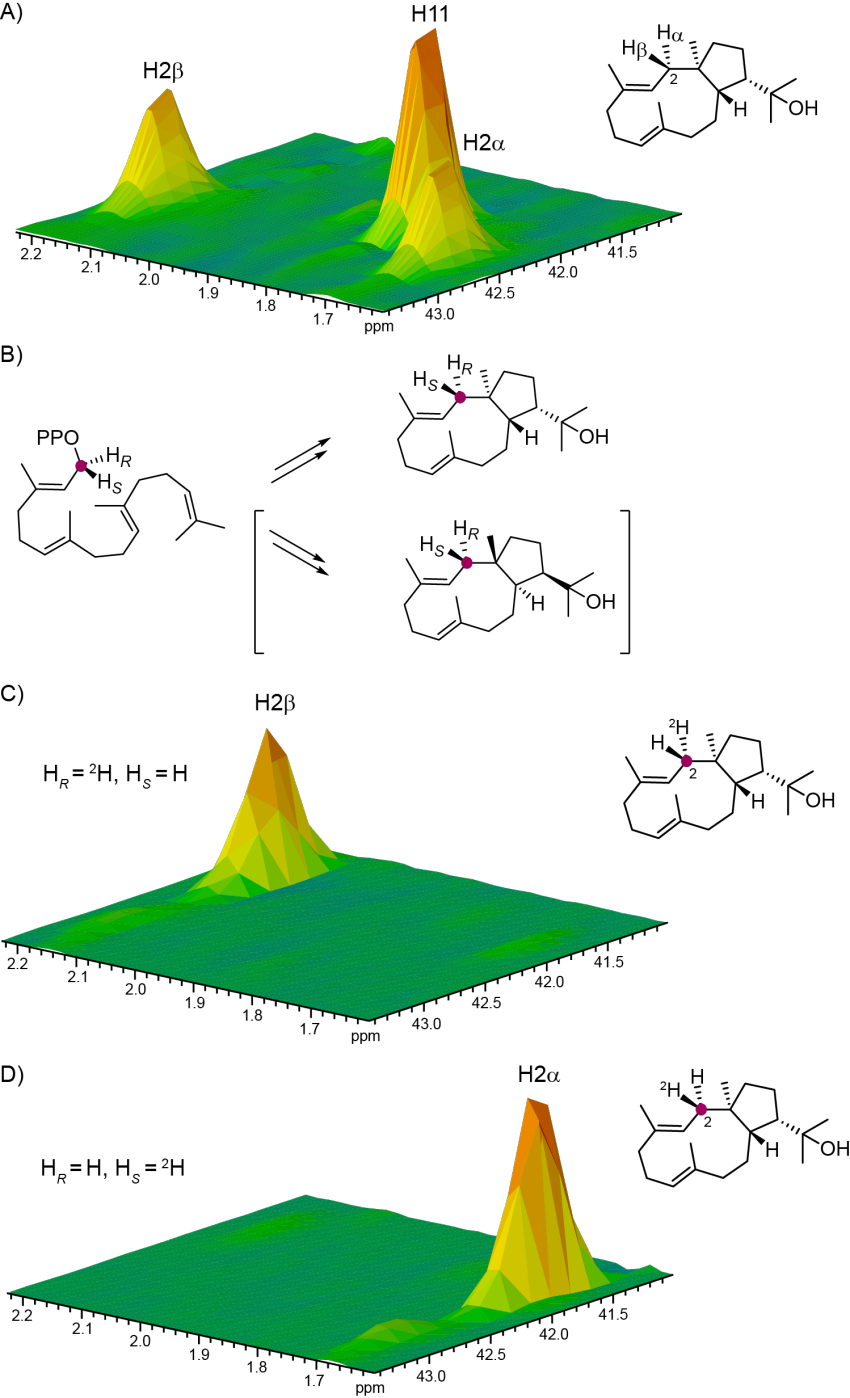
Determination of the absolute configuration of **3**. (A) Partial HSQC spectrum of unlabelled **3** showing the region for C2, (B) cyclisation of GGPP to the two possible enantiomers of **3**, (C) partial HSQC spectrum of the product obtained from (*R*)-(1-^13^C,1-^2^H)GGPP, and (D) partial HSQC spectrum of the product obtained from (*S*)-(1-^13^C,1-^2^H)GGPP. Purple dots indicate ^13^C-labelled carbons.

The proposed cyclisation mechanism from GGPP to **3** is likely a concerted one-step process with 1,11- and 10,14-cyclisation and concomittant attack of water at C15 (Scheme 2). We have recently shown that the absolute configurations of terpenes can be determined by enzymatic conversion of stereoselectively deuterated terpene precursors, because the problem of determining the absolute configuration of the terpene under investigation is simplified to a problem of delineating the relative orientation of its stereocentres to the known absolute configuration at the deuterated carbon [12–13]. This approach was used to determine the absolute configuration of **3** using both enantiomers of (*R*)- and (*S*)-(1-^13^C,1-^2^H)GGPP [14], (*R*)- and (*S*)-(1-^13^C,1-^2^H)FPP, and (*R*)- and (*S*)-(1-^13^C,1-^2^H)GPP [12] in which the additional ^13^C labels were introduced to increase sensitivity in the HSQC analysis of the obtained terpene products. Incubation of (*R*)-(1-^13^C,1-^2^H)GGPP with HdS resulted in the specific incorporation of the deuterium labelling into the 2α position as indicated by a deminished crosspeak in the HSQC spectrum, while the crosspeak for H2β was strongly enhanced because of the ^13^C labelling of C2 (Figure 2). Consistently, the substrate (*S*)-(1-^13^C,1-^2^H)GGPP gave a product with specific incorporation of the deuterium label into the 2β position. Assuming inversion of configuration at C1 for the cyclisation of GGPP to **3** as reported for several other terpene synthases [13,20–22], these findings point to the absolute configuration of (1*R*,3*E*,7*E*,11*S*,12*S*)-18-hydroxydolabella-3,7-diene.

**Figure 3:**
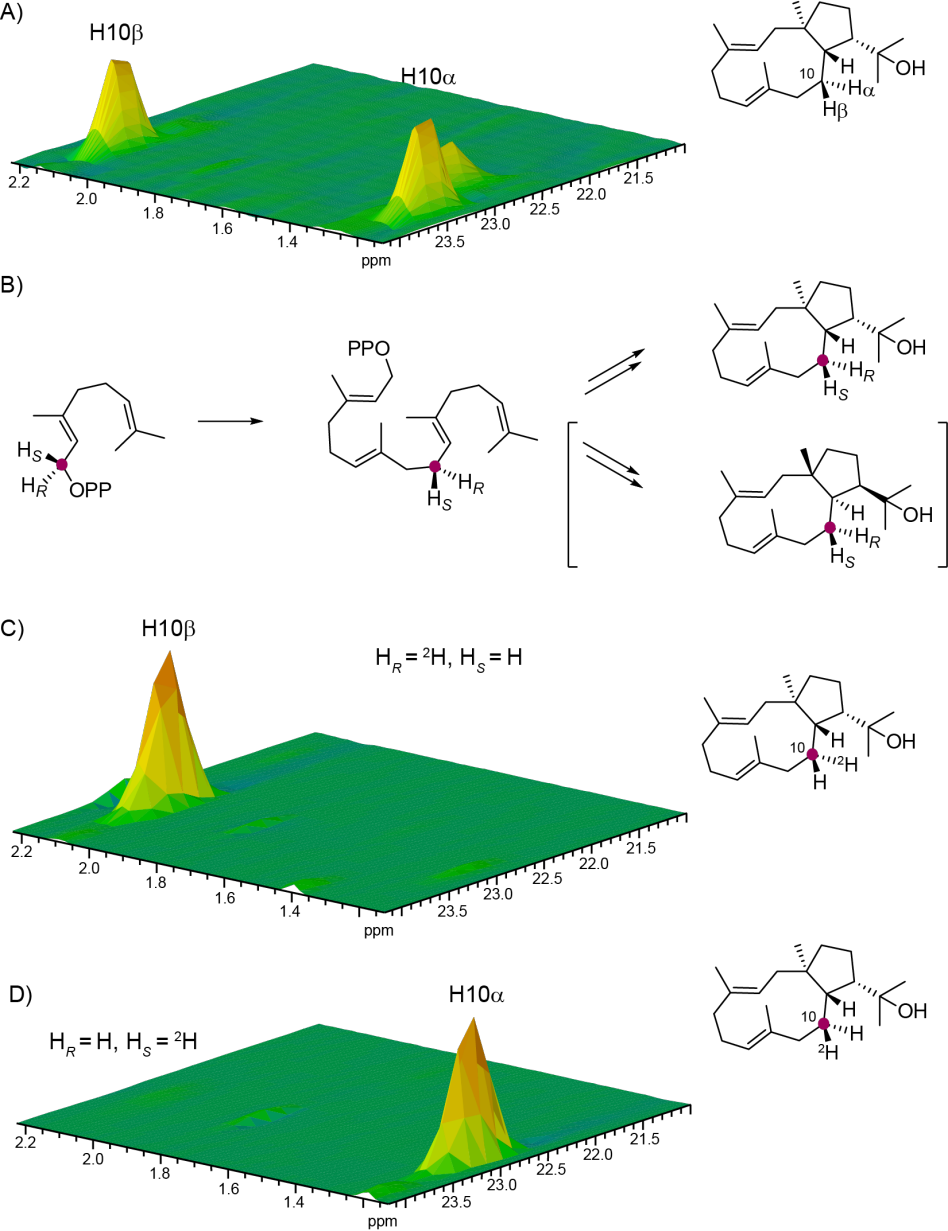
Determination of the absolute configuration of **3**. (A) Partial HSQC spectrum of unlabelled **3** showing the region for C10, (B) elongation of GPP with IPP to GGPP and cyclisation to the two possible enantiomers of **3**, (C) partial HSQC spectrum of the product obtained from (*R*)-(1-^13^C,1-^2^H)GPP, and (D) partial HSQC spectrum of the product obtained from (*S*)-(1-^13^C,1-^2^H)GPP. Purple dots indicate ^13^C-labelled carbons.

For the incubation experiments with (*R*)- and (*S*)-(1-^13^C,1-^2^H)GPP, the terpene monomer IPP, HdS and the GGPP synthase (GGPPS) from *S. cyaneofuscatus* [12] were added to the reaction mixtures for an enzymatic elongation of the GPP isotopomers to the corresponding GGPPs. It is well established that the elongations of oligoprenyl diphosphates with IPP by type I oligoprenyl diphosphate synthases proceeds with inversion of configuration at C1 [23–24]. The conversion of the obtained labelled GGPPs by HdS gave a stereospecific incorporation of the deuterium labelling into H10α from (*R*)-(1-^13^C,1-^2^H)GPP and into H10β from (*S*)-(1-^13^C,1-^2^H)GPP (Figure 3), which pointed to the same absolute configuration for **3** as deduced from the experiments with the two enantiomers of (1-^13^C,1-^2^H)GGPP.

**Figure 4:**
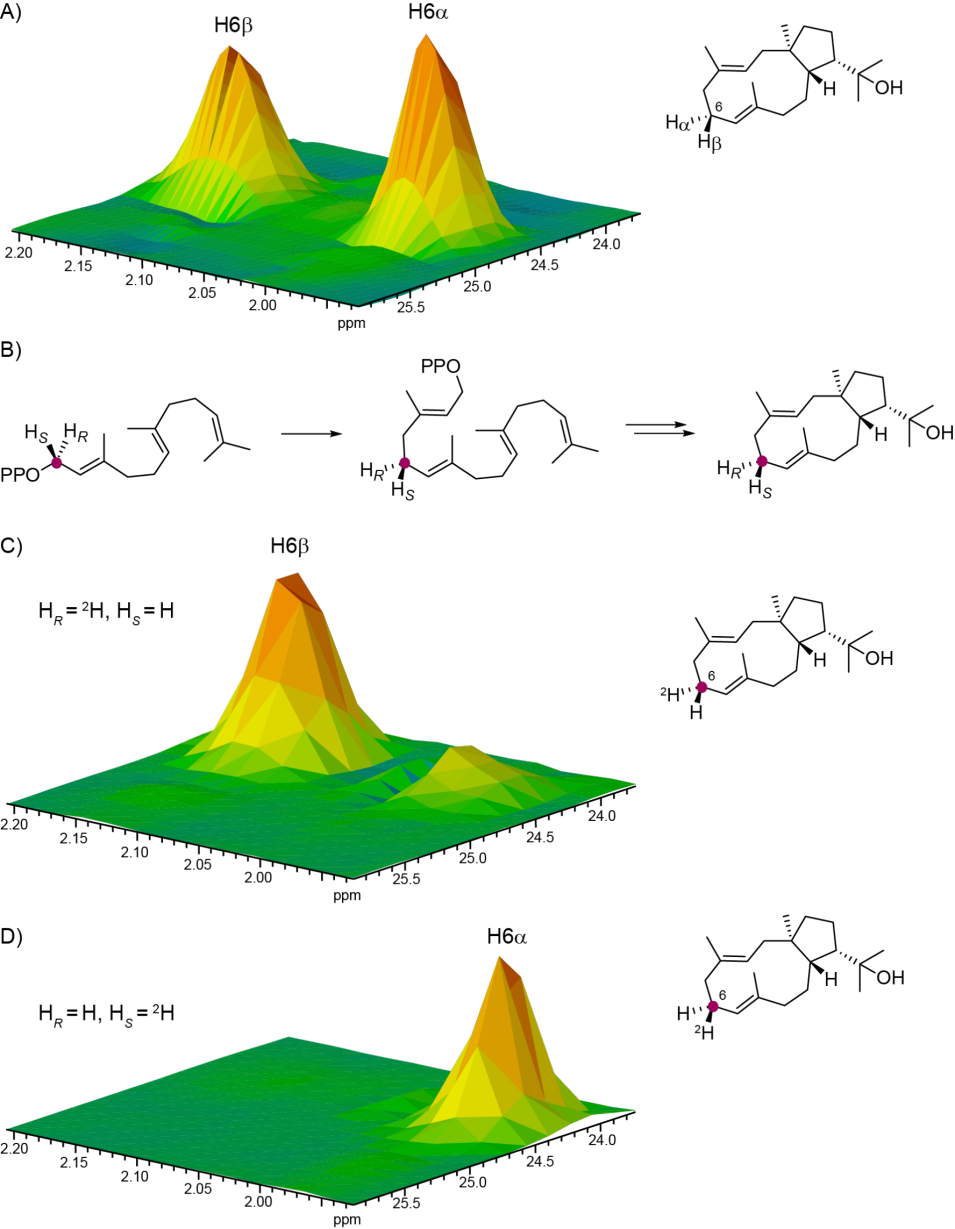
Assignment of H6α and H6β of **3**. (A) Partial HSQC spectrum of unlabelled **3** showing the region for C6, (B) elongation of FPP with IPP to GGPP and cyclisation to **3**, (C) partial HSQC spectrum of the product obtained from (*R*)-(1-^13^C,1-^2^H)FPP, and (D) partial HSQC spectrum of the product obtained from (*S*)-(1-^13^C,1-^2^H)FPP. Purple dots indicate ^13^C-labelled carbons.

Similar incubation experiments were performed with (*R*)- and (*S*)-(1-^13^C,1-^2^H)FPP, IPP, GGPPS and HdS, resulting in the stereospecific incorporation of deuterium labelling into the hydrogens at C6 of **3** (Figure 4). These experiments could not be used to confirm the absolute configuration of the diterpene, because the signals for H6α and H6β could not be unambiguously assigned from the NMR spectra of the unlabelled compound. Instead, the results from these incubation experiments were used for this assignment.

**Figure 5:**
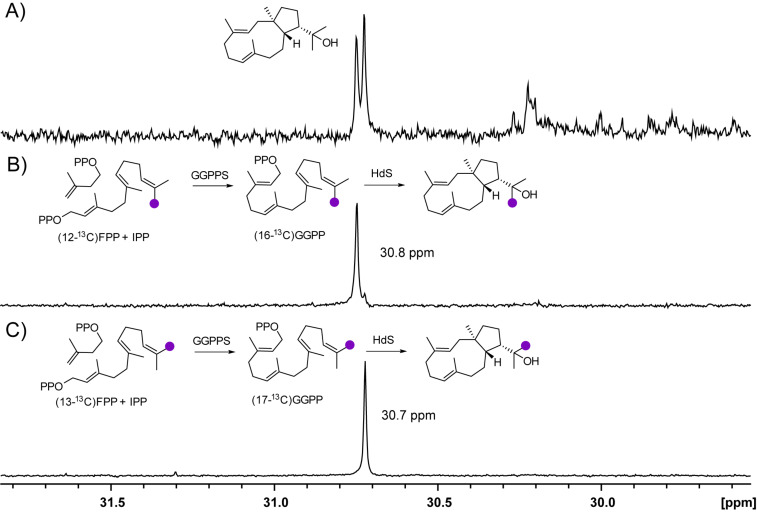
Partial ^13^C NMR spectra of A) unlabeled **3**, B) (^13^C_1_)-**3** arising from incubation of HdS and GGPPS with (12-^13^C)FPP + IPP, and C) (^13^C_1_)-**3** arising from incubation of HdS and GGPPS with (13-^13^C)FPP + IPP. Labelled carbons are indicated by purple dots.

HdS exhibited a defined stereochemical course with respect to the methyl groups in the hydroxyisopropyl group of **3**, as was indicated by conversion of (12-^13^C)FPP and (13-^13^C)FPP [25] with IPP by GGPPS and HdS that resulted in the specific incorporation of labelling into the carbon atoms absorbing at 30.8 ppm and 30.7 ppm, respectively (Figure 5).

### Functional characterisation of bacterial diterpene synthase in planta

**Figure 6:**
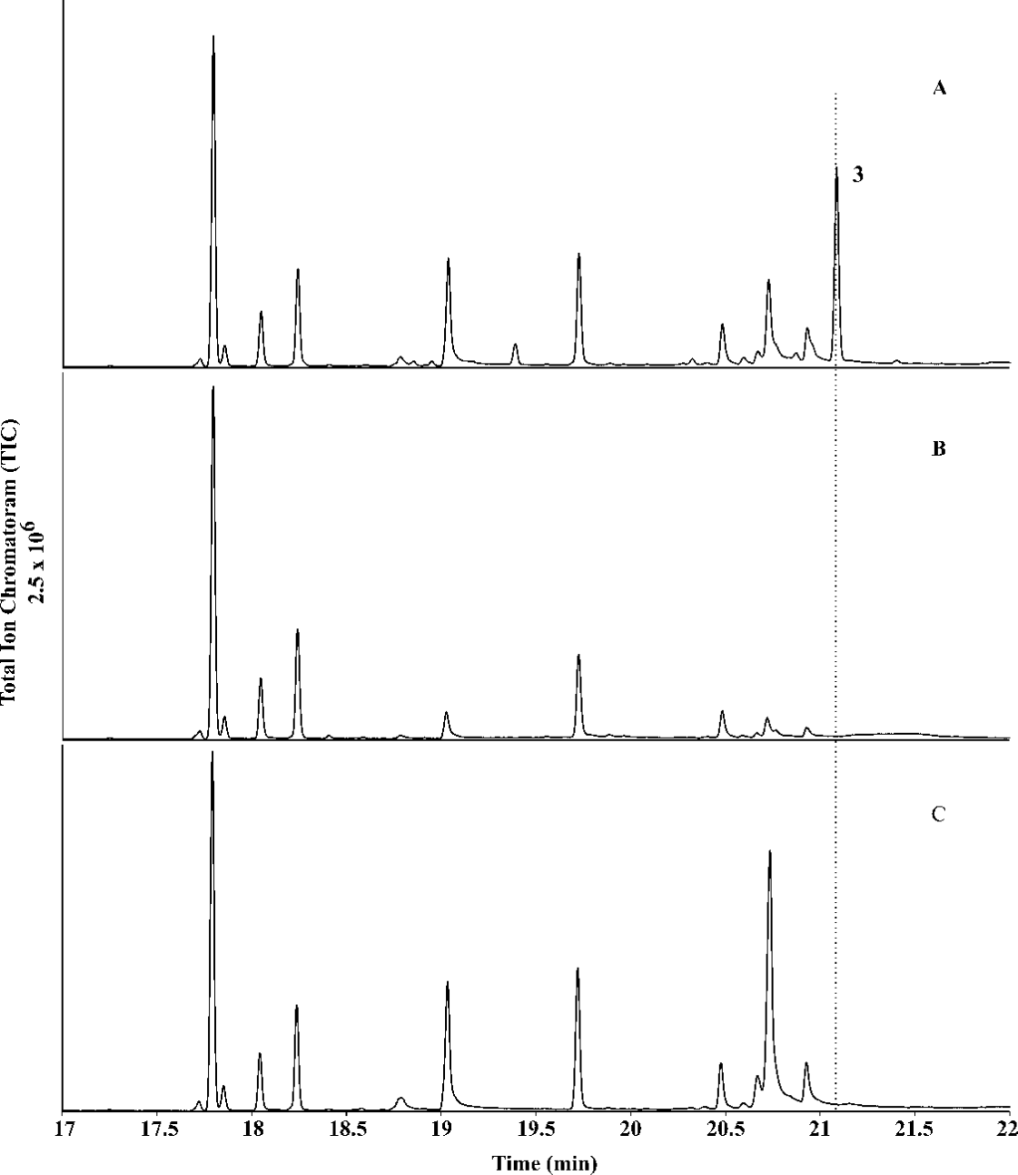
Transient expression of 18-hydroxydolabella-3,7-diene synthase (HdS) in *Nicotiana benthamiana*. Total ion chromatograms of GC–MS analyses of *N. benthamiana* leaf extracts. A) HdS-mit (HdS expressed with mitochondrial targeting signal) showing the production of **3** in planta, B) HdS (expression without targeting signal) and C) empty vector.

To test the catalytic activity of HdS in planta, its corresponding gene was transiently expressed in *N. benthamiana*. Since we have shown before that the mitochondria are a suitable subcellular compartment for the heterologous production of terpenes [26], and it is known that one of the multiple GGPP synthases in plants are targeted to the mitochondria [27], we decided to attempt the expression of HdS with mitochondrial targeting (HdS-mit). A construct without targeting signal (HdS; resulting in cytoplasmic localisation) and an empty vector were used as controls. A *p19* construct [28] was co-infiltrated in all treatments to suppress endogenous silencing of *N. benthamiana* upon agroinfiltration. No difference was found by GC–MS in EtOAc extracts of *N. benthamiana* leaves expressing an empty vector or HdS, while the chromatogram of an extract obtained from HdS-mit expressing leaves revealed an additional major compound (Figure 6). This compound (retention time of 21.08 min) was identified as 18-hydroxydolabella-3,7-diene by GC–MS, using the diterpene alcohol obtained by the in vitro incubations of GGPP with HdS as an authentic standard. A preparative scale isolation of **3** from plant leaves expressing HdS-mit yielded 26.2 mg of the pure diterpene alcohol from 100 g of fresh leaves (0.03% of fresh leaf weight). The obtained material was identical to **3** obtained by in vitro incubation of GGPP with recombinant HdS by ^1^H and ^13^C NMR spectroscopy.

A compound with the same structure as determined from our experiments for (1*R*,3*E*,7*E*,11*S*,12*S*)-18-hydroxydolabella-3,7-diene (**3**), but with different NMR data, was recently reported from the brown alga *Dilophus spiralis* [29]. In this study, a revision for the previously reported structure of (1*S*,3*E*,7*E*,11*R*,12*S*)-18-hydroxydolabella-3,7-diene (1,11-di-*epi*-**3**) for a compound isolated from the brown alga *Dictyota dichotoma* [30] was suggested (Scheme 2C). The same natural product is known from the higher plant *Aglaia odorata* [31], but in this case the reason for the assignment of the reported absolute configuration is unclear, because no optical rotation has been included in this study. It is difficult to judge what the correct structure for the compounds isolated from the brown algae and from *A. odorata* is, but the NMR data and isotopic labelling experiments presented here clearly point to the structure of **3** for the material obtained by us from the diterpene synthase from *C. pinensis*.

## Conclusion

In this study we have reinvestigated a terpene synthase from *Chitinophaga pinensis* that was previously characterised as germacrene A synthase by heterologous expression in *E. coli*. While this result could be reproduced during the course of the present study, the recombinant purified enzyme surprisingly only showed diterpene synthase activity (it did not produce any product from GPP nor FPP) and the obtained product was identified as (1*R*,3*E*,7*E*,11*S*,12*S*)-18-hydroxydolabella-3,7-diene. Notably, heterologous expression in the plant *Nicotiana benthamiana* and targeting to the mitochondria resulted in the production of the same diterpene alcohol. Although the mitochondria of *N. benthamiana* also produce FPP [32], again no germacrene D was detected. Taken together, these experiments demonstrate that the expression of one and the same terpene synthase in different organisms may lead to the formation of different products and even an altered substrate specificity. Indeed, it has been shown before that small alterations in the conditions such as a change of the metal cofactor can result in a switch from FPP to GPP synthase activity for an oligoprenyl diphosphate synthase from the beetle *Phaedon cochleariae* [33]. Similar small changes of the conditions, e.g., of the pH or the presence of different metal cofactors, may also change the product profile of a terpene synthase in different heterologous hosts. Changes in the product profile of terpene synthases depending on the host that was used to express the gene have been reported by Ginglinger et al., who have shown that *Arabidopsis* TPS10 produced mainly linalool when expressed in yeast and *N. benthamiana*, while the *E. coli* expressed protein catalysed the formation of mainly β-myrcene and β-ocimene [34]. The authors suggested different cofactor availabilities and biochemical conditions in the different hosts as the reason for their findings. Also Fischer et al. pointed out the effect that the host can have on the product specificity of terpene synthases [35]. In this context substrate availability is another issue to be considered: While no GGPP synthase is known in *E. coli*, this diterpene precursor is produced in the mitochondria of *N. benthamiana*. The yield of 18-hydroxydolabella-3,7-diene in planta of 26.2 mg per 100 g of fresh leaves is useful for the preparative scale production of the diterpene alcohol that can easily be isolated by extraction and column chromatography, which underpins the potential of plants, besides the recently reviewed microbial hosts for the sustainable production of diterpenes [36], as expression systems for secondary metabolite genes. The function of the investigated terpene synthase from *C. pinensis* in its natural context remains elusive, since neither (1*R*,3*E*,7*E*,11*S*,12*S*)-18-hydroxydolabella-3,7-diene nor germacrene A or its Cope rearrangement product β-elemene could be detected in laboratory cultures [37].

## Supporting Information

File 1 Experimental details for gene expression and enzyme incubation experiments, NMR spectra of (1*R*,3*E*,7*E*,11*S*,12*S*)-18-hydroxydolabella-3,7-diene, and heterologous expression in *Nicotiana benthamiana*.
