# Supplementary material for: 18-Hydroxydolabella-3,7-diene synthase – a diterpene synthase from Chitinophaga pinensis
Source: Beilstein J Org Chem. 2017 Aug 23;13:1770–80. doi: 10.3762/bjoc.13.171 (PMC5588592; doi:10.3762/bjoc.13.171)
Supplement: File 1 — Experimental details for gene expression and enzyme incubation experiments, NMR spectra of (1R,3E,7E,11S,12S)-18-hydroxydolabella-3,7-diene, and heterologous expression in Nicotiana benthamiana. [file Beilstein_J_Org_Chem-13-1770-s001.pdf]

Supporting Information

for

18-Hydroxydolabella-3,7-diene synthase – a diterpene synthase from *Chitinophaga pinensis*

Jeroen S. Dickschat[1]*, Jan Rinkel[1], Patrick Rabe[1], Arman Beyraghdar Kashkooli[2] and Harro J. Bouwmeester[3]

Address: [1]Kekulé-Institute of Organic Chemistry and Biochemistry, University of Bonn, Gerhard-Domagk-Straße 1, 53121 Bonn, Germany, [2]Laboratory of Plant Physiology, Wageningen University, Droevendaalsesteeg 1, 6708 PB Wageningen, The Netherlands, and [3]Swammerdam Institute for Life Sciences, University of Amsterdam, Sciencepark 904, 1098 XH Amsterdam, The Netherlands

Email: Jeroen S. Dickschat - dickschat@uni-bonn.de

*Corresponding author

Experimental details for gene expression and enzyme incubation experiments, NMR spectra of (1*R*,3*E*,7*E*,11*S*,12*S*)-18-hydroxydolabella-3,7-diene, and heterologous expression in *Nicotiana benthamiana*

Gene cloning

Cloning of the HpS gene (accession number: WP012789469, old accession number: YP003121494) into the expression vector pET28c was reported previously [1].

Gene expression and protein purification

The *E. coli* BL21 transformant carrying the expression vector pET28c_WP012789469 was used to inoculate an overnight preculture at 37 °C in LB medium (10 mL; 10 g/L tryptone, 5 g/L yeast extract, 5 g/L NaCl; pH 7.0) containing kanamycin (50 mg/L). The grown culture was used to inoculate an LB main culture (1/1000) that was incubated at 37 °C to reach OD_{600} = 0.4–0.6. The cultures were cooled to 18 °C and expression was induced by IPTG (0.4 mM). After 18 h, the cells were centrifuged and resuspended in TRIS binding buffer (10 mL/L culture; 50 mM TRIS, 0.5 M NaCl, 20 mM imidazole, 1 mM $MgCl_2$, pH 7.0). Cell disruption was carried out using an ultra sonotrode on ice (5 × 1 min), before centrifugation of the cell debris (11000 rpm, 4 °C) to yield the soluble protein fraction. The desired protein was purified by Ni-NTA affinity chromatography (Qiagen), washed with binding buffer (2 × 10 mL/L culture) and desorbed from the resin by TRIS elution buffer (10 mL/L culture; 50 mM TRIS, 0.5 M NaCl, 0.5 M imidazole, 1 mM $MgCl_2$, pH 7.0). The obtained fractions were checked by SDS-PAGE (Figure S2) and the obtained pure fractions (1.5 mg/mL, determined by Bradford assay) were used for incubation experiments.

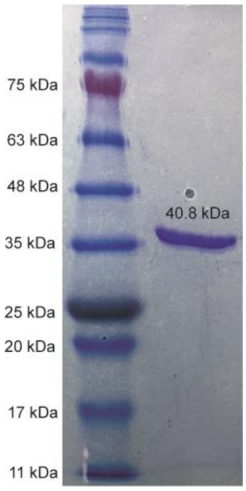

Figure S1: SDS-PAGE analysis of HdS-elution fraction (right).

Repeated headspace analysis using CLSA

In our previous report [1] we have described the production of terpenes emitted by *E. coli* BL21 cultures, carrying a pET28c derived plasmid for the expression of the cloned HpS gene. Under these conditions we have obtained sesquiterpene products, but in the present work in vitro experiments with the purified recombinant enzyme only yielded a product from the diterpene precursor GGPP, but no products from the sesquiterpene precursor FPP. To clarify the situation and to exclude, e.g., a confusion of materials, we have repeated the previous experiment. Therefore, *E. coli* BL21 transformed with the pET28c expression construct with the cloned HpS gene was again grown at 37 °C to OD_{600} = 0.4 and then cooled to 18 °C. Gene expression was induced with IPTG (0.4 mM) and the culture was directly connected to a CLSA (closed loop stripping apparatus) [2] for capturing volatiles emitted by the bacterial culture on a charcoal filter trap. Volatiles were collected for 16 h at 18 °C. The charcoal filter was extracted with CH_2Cl_2 and the extract was directly analysed by GC–MS. The analysis showed a similar result as reported previously, with the main products (with increasing retention times in Figure S1) β-elemene, γ-elemene, β-selinene, and α-selinene. The reason for the different outcomes in the in vivo and the

in vitro experiments is unclear, but the repeated analysis shows reproducibility of these results and excludes any confusion of materials.

GC–MS analyses

GC–MS analyses were carried out on a 7890B GC connected to a 5977A mass detector (Agilent) using a HP5-MS fused silica capillary column (30 m, 0.25 mm i. d., 0.50 μm film). GC parameters were 1) inlet pressure: 77.1 kPa, He at 23.3 mL min^{-1}, 2) injection volume: 2 μL, 3) temperature program: 5 min at 50 °C increasing at 5 °C min^{-1} to 320 °C, 4) split ratio: 10:1, 60 s valve time, and 5) carrier gas: He at 1.2 mL min^{-1}. MS parameters were 1) source: 230 °C, 2) transfer line: 250 °C, 3) quadrupole: 150 °C and 4) electron energy: 70 eV. Retention indices (*I*) were determined in comparison to a homologous series of *n*-alkanes (C_7–C_{40}).

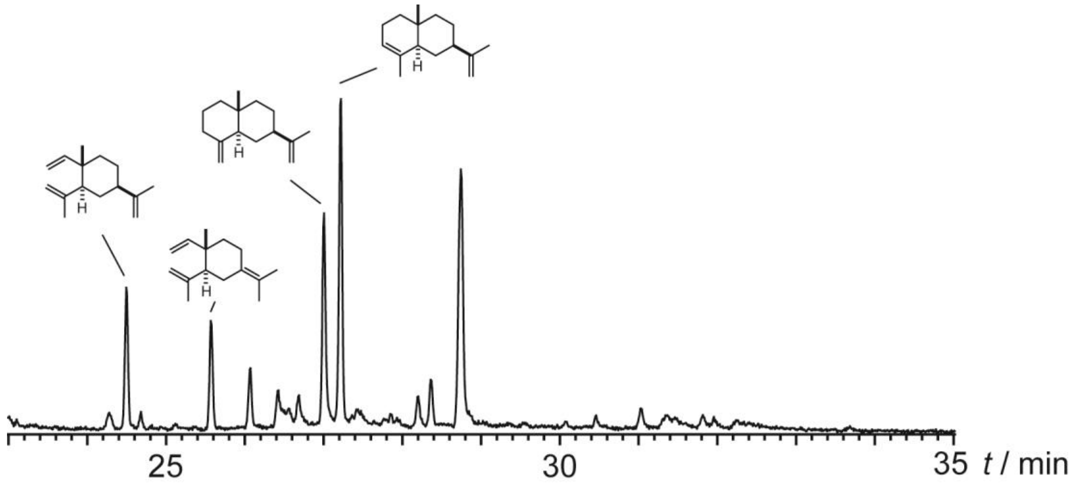

Figure S2: Total ion chromatogram of a headspace extract from transformed *E. coli* BL21.

Preparative scale isolation of enzyme product in vitro

A solution of GGPP (20 mg) in binding buffer (50 mL) was added to the HdS elution fraction arising from 8 L culture. The enzyme reaction was incubated overnight at 28 °C and extracted with pentane (3 × 30 mL). The obtained organic extracts were dried with $MgSO_4$ and concentrated under reduced pressure to give a crude product (4.5 mg), which was purified using silica gel chromatography [pentane:Et_2O (5:1)] to yield **3** as a colorless oil (1.3 mg).

(1*R*,3*E*,7*E*,11*S*,12*S*)-18-Hydroxydolabella-3,7-diene (**3**): R_f [cyclohexane:EtOAc (3:1)] = 0.64. GC (HP5-MS): *I* = 2162. HRMS (QTOF): *m*/*z* = 290.2586 (calc. for $[C_{20}H_{34}O]^+$ 290.2604). $[\alpha]_D^{20}$ = +23.8 (*c*=0.86, (^{2}H)chloroform). IR (diamond ATR): $\tilde{\nu}$ = 3359 (br, m), 2969 (s), 2940 (s), 2867 (s), 1448 (s), 1372 (s), 1314 (m), 1222 (m), 1147 (m), 1081 (m), 1017 (s), 943 (s), 914 (m), 866 (s), 768 (m), 660 (w), 538 (m), 496 (m), 443 (w), 421 (w) cm^{-1}. NMR data are presented in Table 1.

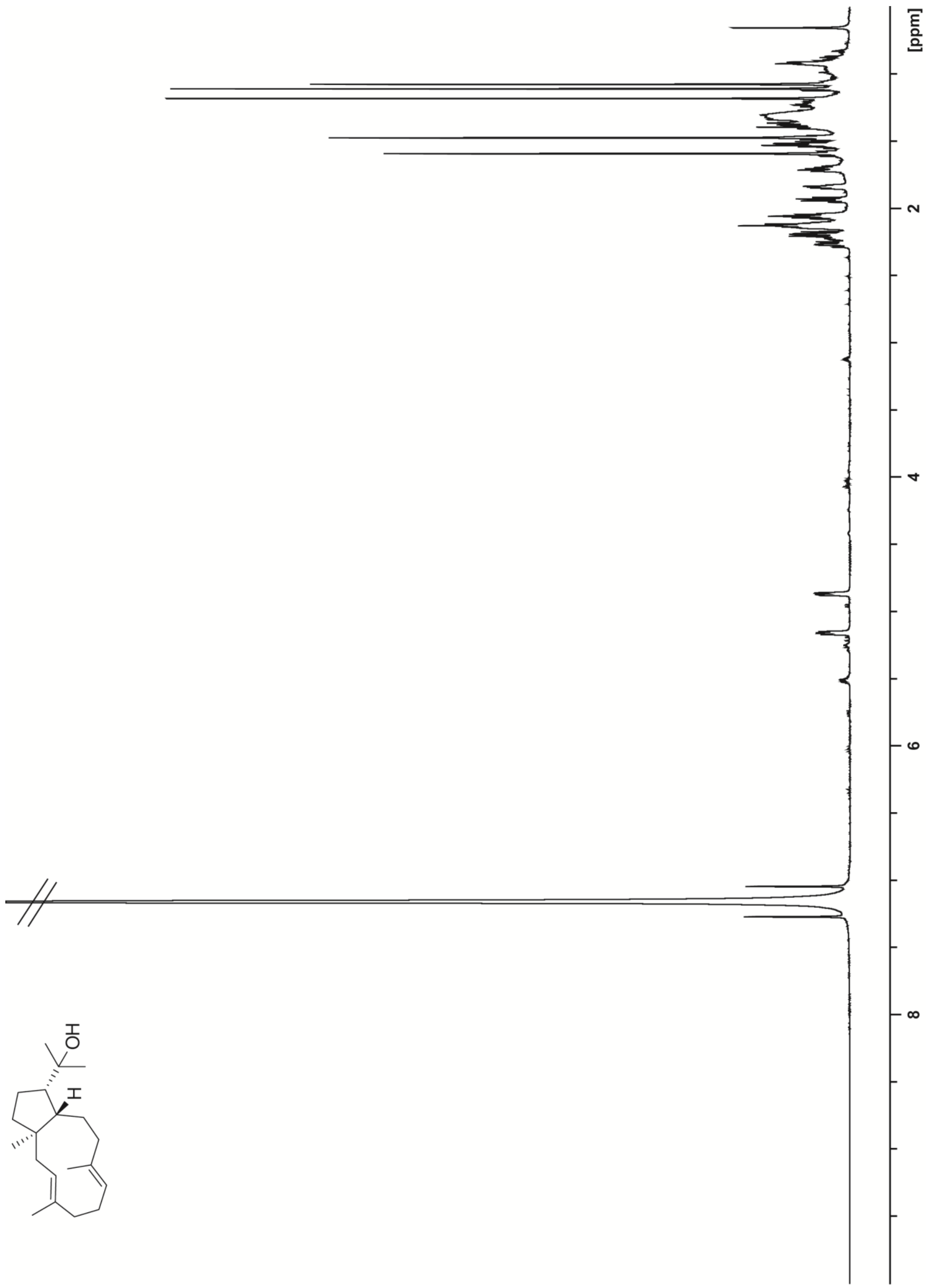

Figure S3: ^{1}H NMR spectrum (700 MHz) of **3** in C_6D_6.

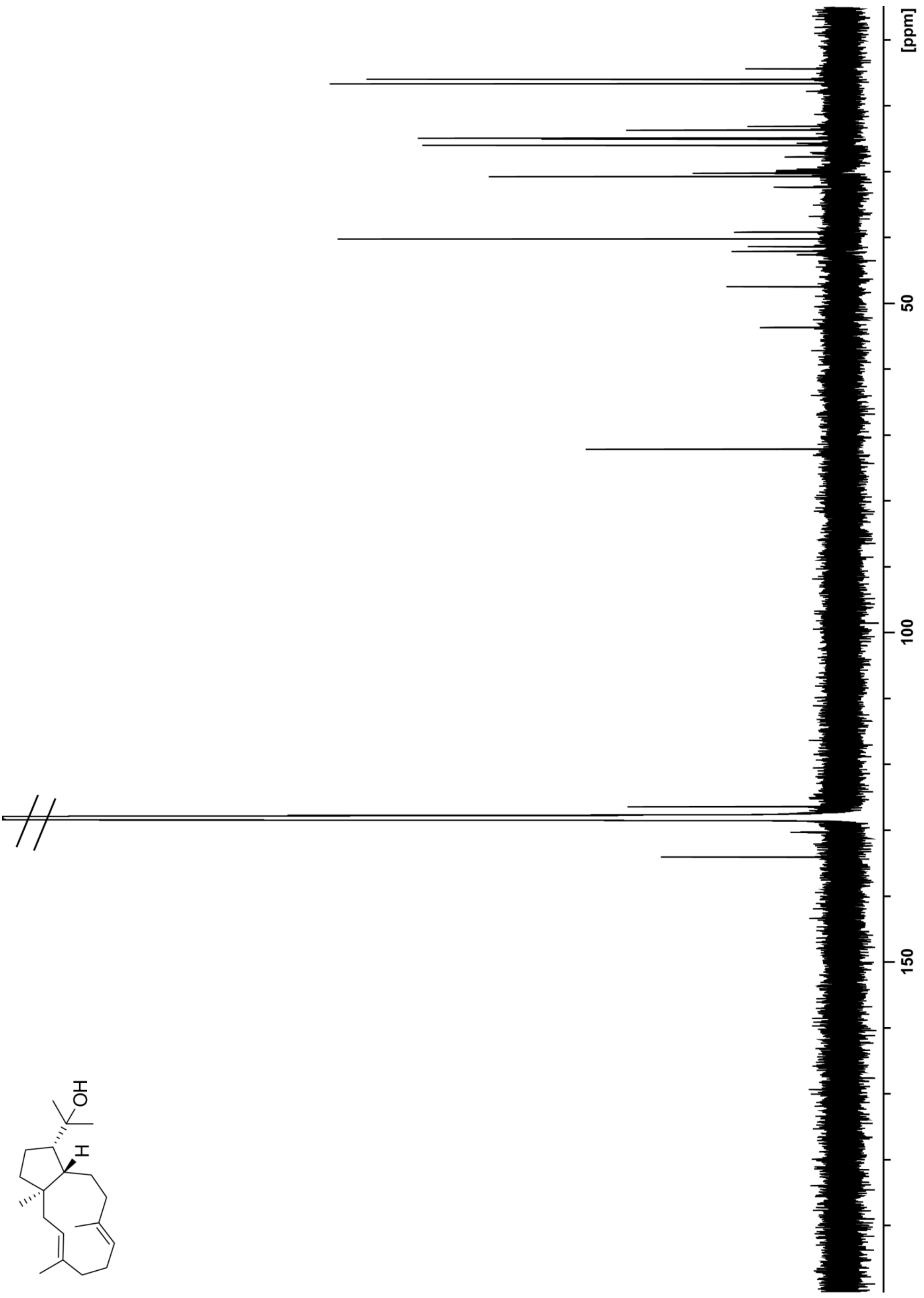

Figure S4: ^{13}C NMR spectrum (176 MHz) of **3** in C_6D_6. Please note that the ^{13}C NMR shows signals for different conformers of the macrocycle.

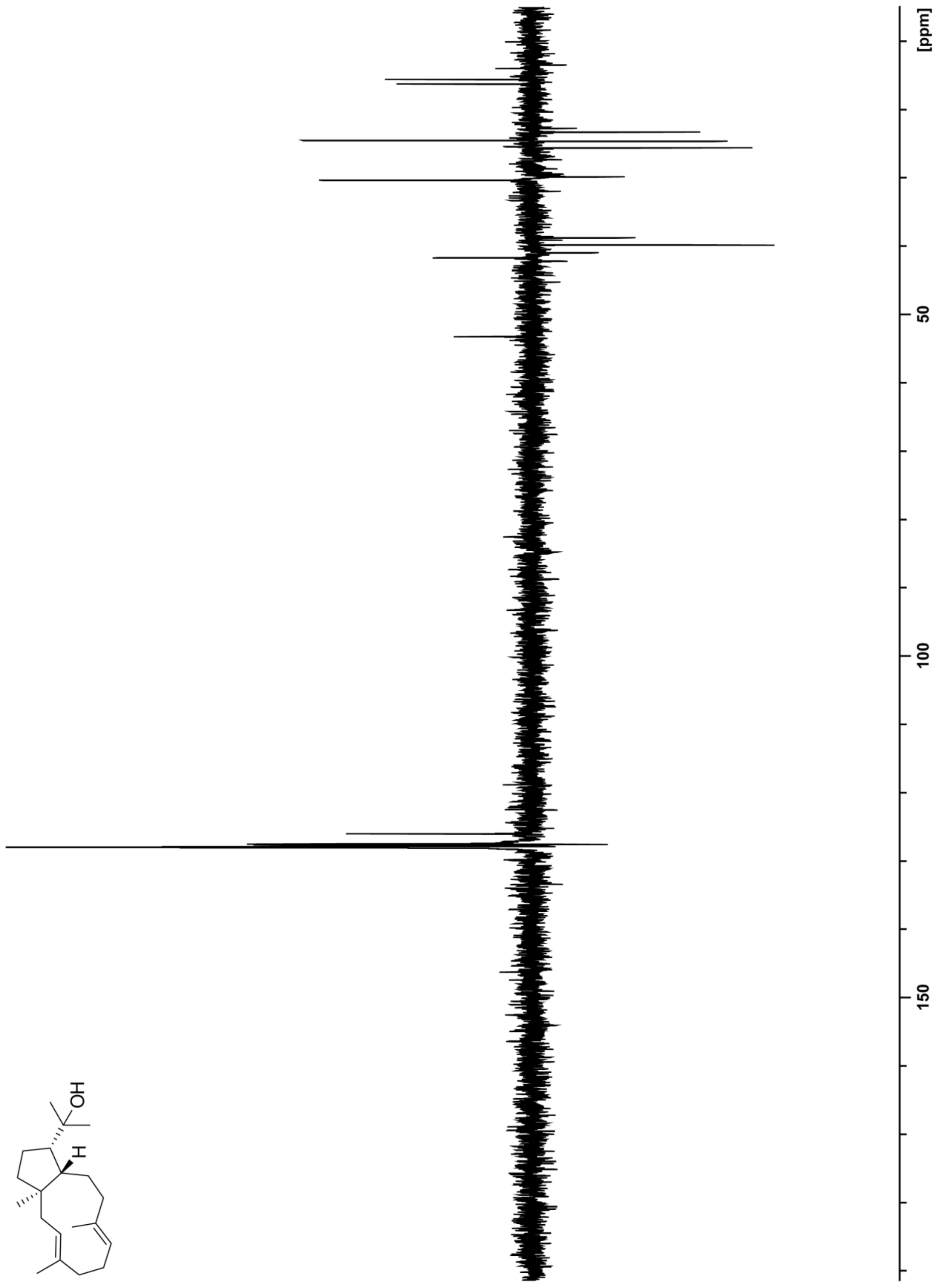

Figure S5: DEPT-135 NMR spectrum (176 MHz) of **3** in C_6D_6.

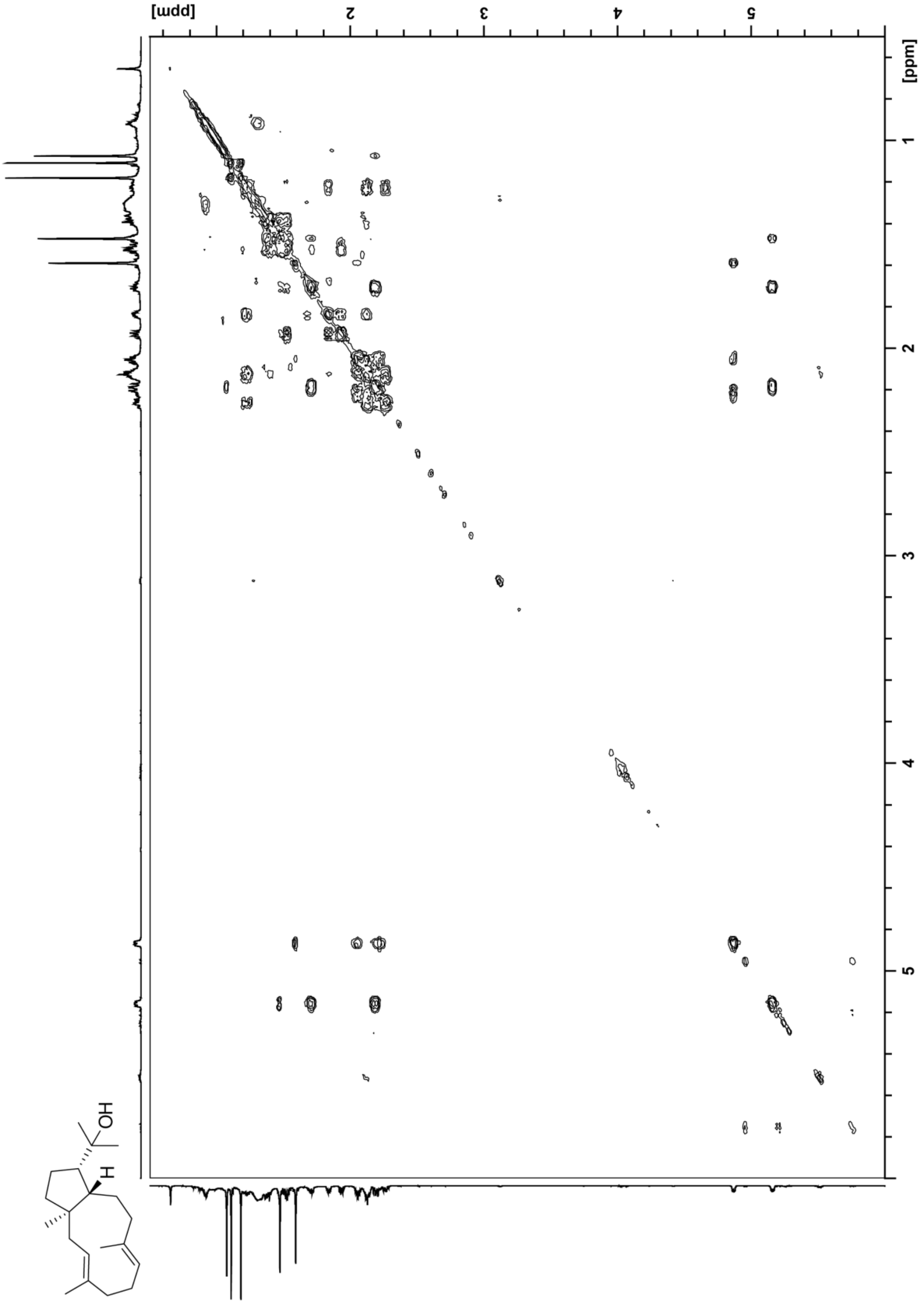

Figure S6: $^{1}H,^{1}H$-COSY spectrum (700 MHz) of **3** in C_6D_6.

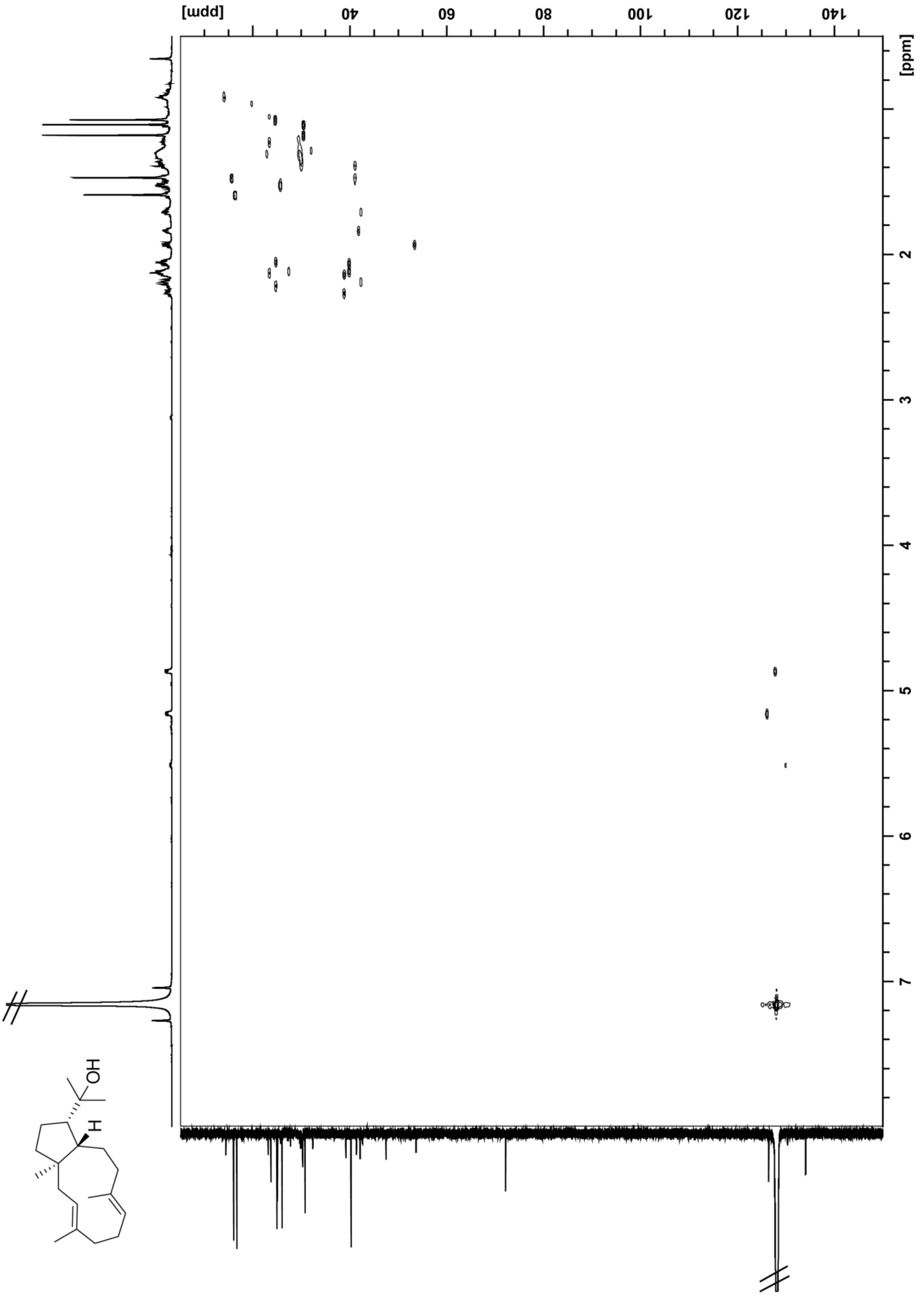

Figure S7: $^{1}H,^{13}C$-HSQC spectrum (700 MHz) of **3** in C_6D_6.

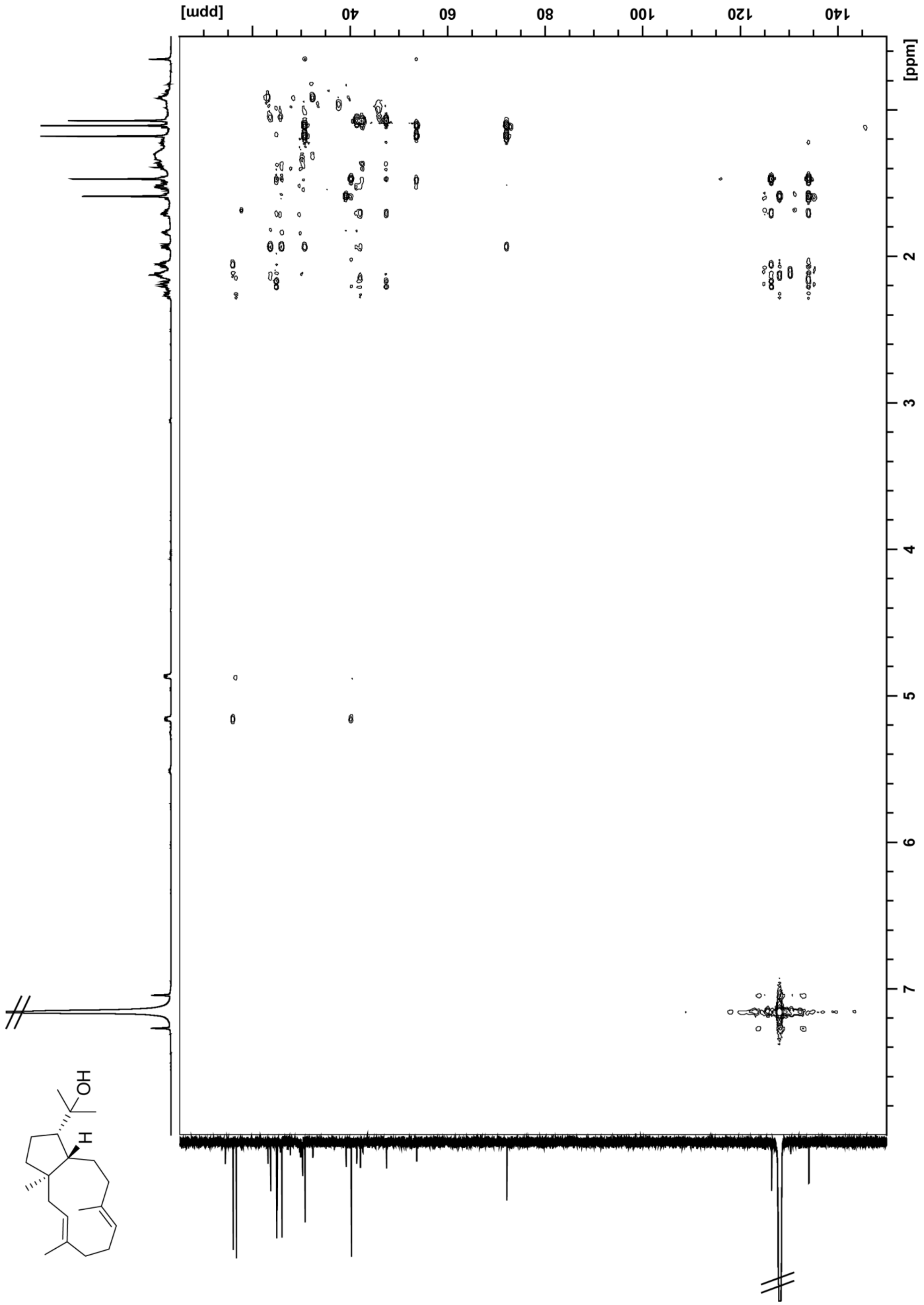

Figure S8: ^{1}H,^{13}C-HMBC spectrum (700 MHz) of **3** in C_6D_6.

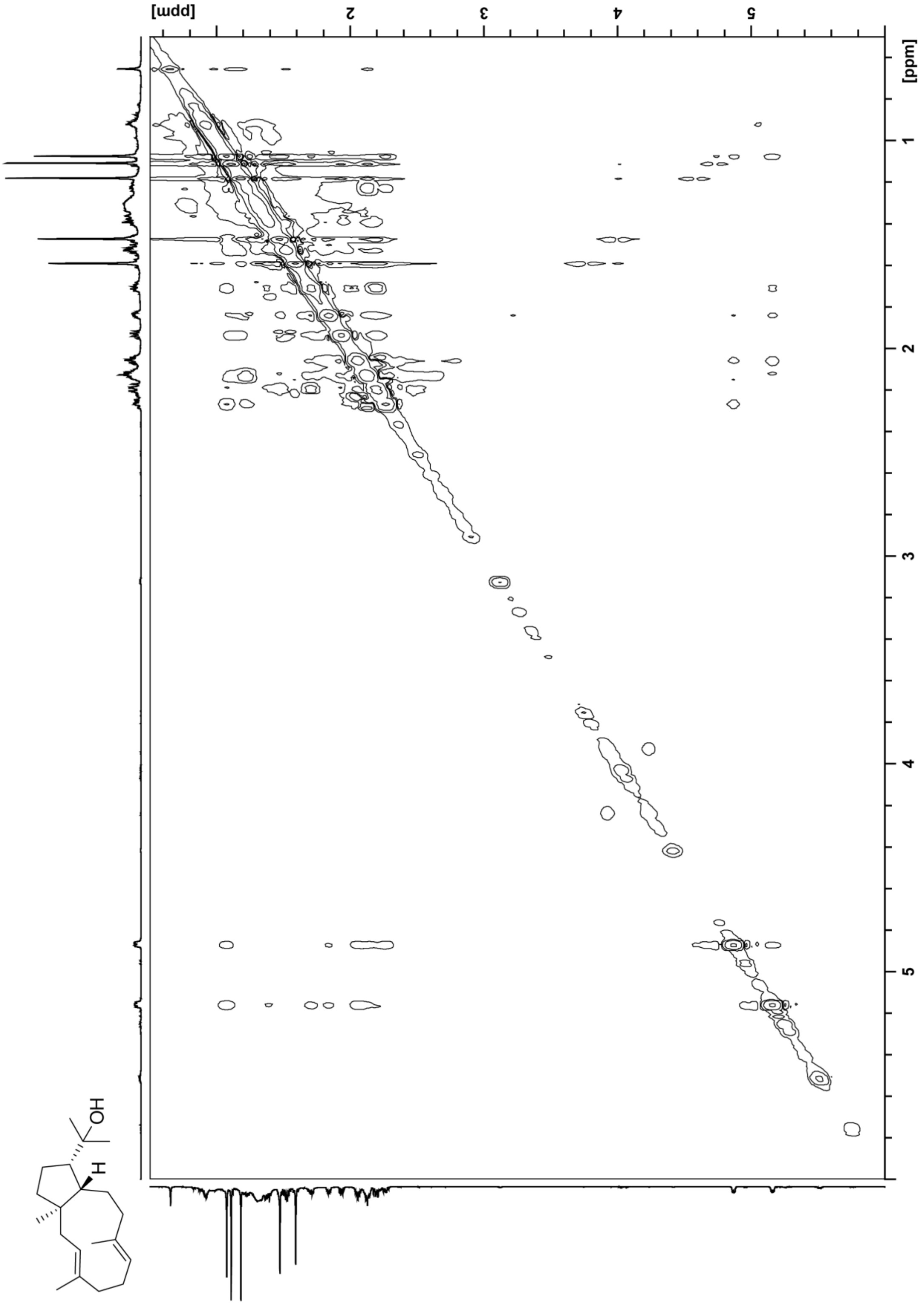

Figure S9: ^{1}H,^{1}H-NOESY spectrum (700 MHz) of **3** in C_6D_6.

Incubation experiments using isotopically labeled substrates

The substrates (1*R*)- and (1*S*)-(1-^{13}C,1-^{2}H)GPP, (1*R*)- and (1*S*)-(1-^{13}C,1-^{2}H)FPP and (1*R*)- and (1*S*)-(1-^{13}C,1-^{2}H)GGPP (1 mg each) or (12-^{13}C) and (13-^{13}C)FPP (0.5 mg each) were dissolved in aqueous NH_4HCO_3 solution (25 mM, 1 mL) and added to incubation buffer (4 mL; 50 mM TRIS, 10 mM $MgCl_2$, 20% glycerol, pH 8.4). A solution of IPP (1 mL; 1 mg/mL in 25 mM NH_4HCO_3) was added to the GPPs and FPPs as well as 2 mL GGPP synthase [3] (*S. cyaneofuscatus*) elution fraction. To start the terpene cyclisation, HdS elution fraction (4 mL) prepared as described above was added to each of the eight samples and the reaction was incubated for 3 h at 28 °C. Extraction was carried out with C_6D_6 (0.65 mL and 0.3 mL) and the organic extracts were analyzed by GC–MS and NMR.

Plasmid construction for gene expression in *Nicotiana benthamiana*

The coding sequence (CDS) of HdS was amplified from a pET28c vector (Novagen) by add□ition of NcoI and NotI restriction sites. The forward primer sequence used for amplification of CDS was 5’ CCATGGTGTTGTATCCTACATTCAGCATT 3’ and the reverse primer 5’ GCGGCCGCTTACGCGTAAACCTGCTGC 3’. A PCR fragment of the expected size (≈1000 bp) was amplified by a proofreading Q5 DNA polymerase (NEB). The obtained fragment was cloned into pCR8/GW/TOPO (Invitrogen) and sequenced with M13 sequencing primers. Plasmid DNA (1 µg) was digested with 10 units of NcoI and NotI. Then the digested fragment was ligated into ImpactVector1.1 (http://www.impactvector.com/) to fuse it with the Rubisco promoter. ImpactVector1.5 was used to fuse the fragment to a Rubisco promoter and the CoxIV mitochondrial targeting signal. Subsequently, an LR reaction, using Gateway LR Clonase II Enzyme Mix, was performed to clone the obtained constructs between the T-DNA right and left borders of the plant binary vector, pBINPlus [4]. Finally, the constructs

were transformed into *Agrobacterium tumefaciens* strain AGL0 for transient plant transformation.

Transient heterologous expression in *Nicotiana benthamiana*

In planta expression of HdS was done by *Agrobacterium* mediated transformation. Bacteria were grown at 28 °C for 40 h in a shaker incubator (250 rpm) with addition of rifampicin (37 µg mL^{-1}) and kanamycin (50 µg mL^{-1}), starting from a single confirmed colony. Cells were then collected by centrifugation at 3500*g* for 15 min. The medium was discarded and the pellet was resuspended in an agroinfiltration buffer (10 mM MES, 10 mM $MgCl_2$ and 100 µM acetosyringone) to get an OD_{600} of 0.5. Bacteria batches containing different constructs (p19 and the different HdS constructs) were carefully mixed on a roller mixer for 150 min before agroinfiltration. The agrobacteria were injected with a 1 mL syringe (without needle) to the abaxial side of the leaves of 4 weeks old *N. benthamiana* plants. Transformed plants were kept for 4 days in the greenhouse (day-night temperature of 22°C–20°C and a day-night rhythm of 16 h light–8 h dark).

Metabolites extraction and GC–MS analysis

Four days post infiltration, fresh leaves (500 mg) of each transformant (HdS-mit, HdS or empty vector) were harvested and frozen in liquid N_2. The leaves were ground to a fine powder and extracted with ethyl acetate (4 × 2 mL). The combined extracts were centrifuged at 4 °C (1000*g*) and supernatant was collected. The extracts were dried with anhydrous Na_2SO_4 and concentrated to a residual volume of 0.5 mL for GC–MS analysis.

Preparative scale isolation from plant material

Small cut leaves (100 g) were refluxed in EtOAc (250 mL) for 4 h. The extract was filtrated and the residue was again mixed with EtOAc (200 mL) and treated by ultrasonification (4 × 15 min, 100%). The extracts were combined, dried with $MgSO_4$ and purified by repeated silica gel column chromatography [pentane/Et_2O (5:1)] to yield **3** as a colorless oil (26.2 mg, 0.03% of fresh leaf weight).

References

1. Rabe, P.; Dickschat, J. S. *Angew. Chem. Int. Ed.,* **2013,** *52,* 1810–1812.

2. Grob, K.; Zürcher, F. *J. Chromatogr. A,* **1976,** *117,* 285–294.

3. Rabe, P.; Rinkel, J.; Dolja, E.; Schmitz, T.; Nubbemeyer, B.; Luu, T. H.; Dickschat, J. S. *Angew. Chem. Int. Ed.,* **2017,** *56,* 2776–2779.

4. van Engelen, F. A.; Molthoff, J. W.; Conner, A. J.; Nap, J.-P.; Pereira, A.; Stiekema, W. J. *Transgenic Res.,* **1995,** *4,* 288–290.
